# Leprosy relapse after multidrug therapy: Systematic review and meta-analysis

**DOI:** 10.1371/journal.pntd.0011870

**Published:** 2025-10-13

**Authors:** Fabiane Veronica da Silva, Gutembergue Santos de Sousa, Eric Brito Ferraz, Pãmela Rodrigues De Souza Silva, Juliana Akie Takahashi, Omar Ariel Espinosa, Jack Roberto Silva Fhon, Roberta Olmo Pinheiro, Vilanice Alves de Araújo Püschel, Zélia Ferreira Caçador Anastácio, Silvana Margarida Benevides Ferreira

**Affiliations:** 1 Graduate Program in Nursing at the Federal University of Mato Grosso, Avenida Fernando Correia, Cuiabá, Mato Grosso, Brazil; 2 JBI Brazilian Centre for Evidence-based Practice - JBI Brazil, São Paulo, São Paulo, Brazil; 3 Surgical-Medical Department, School of Nursing at the University of São Paulo, São Paulo, São Paulo, Brazil; 4 Library Wanda de Aguiar Horta, School of Nursing at the University of São Paulo, São Paulo, São Paulo, Brazil; 5 Department of Genomics, National Center for Medical Genetics and Genomics, Caja de Seguro Social, Panamá, Panamá,; 6 Nursing School at the University of São Paulo, Avenida Doutor Enéas Carvalho de Aguiar, São Paulo, São Paulo, Brazil; 7 Leprosy Laboratory, Oswaldo Cruz Institute, Oswaldo Cruz Foundation, Rio de Janeiro, Brazil; 8 Research Center on Child Studies, University of Minho, Campus de Gualtar, Braga, Portugal; Universidade Federal do Para, BRAZIL

## Abstract

**Objective:**

To synthesize the scientific evidence regarding the prevalence of leprosy relapse following multidrug therapy.

**Method:**

A systematic review was conducted following the JBI methodology for prevalence studies and reported according to the guidelines, with the registration number CRD42020177141. The inclusion criteria were based on the mnemonics (Population, Condition, Context). Population: Individuals of any age or sex diagnosed with leprosy relapse and previously treated with paucibacillary or multibacillary multidrug therapy. Conditions: Leprosy relapse after multidrug therapy, measured as the proportion of cases. Context: Studies conducted within the healthcare service settings. The databases searched included Medline, Latin American and Caribbean Health Sciences Literature (LILACS), Embase, Cumulative Index to Nursing and Allied Health Literature (CINAHL), Scopus, WoS, and Caribbean Public Health Agency (CARPHA). The references were managed using Mendeley. A random-effects meta-analysis model was employed, and heterogeneity was assessed using Higgins’ I² statistics.

**Results:**

Of 26 studies (a combined sample of 71,385 participants), 19 were included in the meta-analysis. A higher prevalence of relapse was observed in working-age males, multibacillary cases with a high bacillary load, and those with established physical disabilities. The estimated prevalence of relapse across studies ranged from 0% to 10%, with a pooled estimate of 4% in India (95% CI: 0.03–0.05). The overall point estimate for relapse using regular multidrug therapy was 0.04 (95% CI: 0.02–0.05).

**Conclusion:**

The prevalence of relapse varied according to the geographic location and type of multidrug therapy, with substantial heterogeneity across studies. These findings suggest that factors such as individual patient characteristics, treatment adherence, and capacity for healthcare services may have influenced the outcomes observed in this review.

## Author summary

Leprosy is considered a millennial and neglected disease. Despite the treatment with multidrug therapy (MDT) adopted by the World Health Organization (WHO) having reduced the number of new cases of the disease, it remains a public health problem. The physical disabilities associated with late diagnosis, stigma, discrimination, social restrictions, and consequently the loss of quality of life can increase the disease’s prevalence. Another important issue that negatively impacts disease control is the occurrence of relapse after MDT, which can compromise therapeutic efficacy. The causes of relapse are commonly associated with therapeutic failure due to incomplete treatment, errors in operational classification, and the existence of resistant bacteria. This study aims to synthesize the evidence on the prevalence of relapse after the adoption of MDT, which could contributed to the implementation of more assertive strategies to prevent the disease.

## Introduction

Leprosy (Hansen’s disease) is an ancient, neglected infectious disease caused by *Mycobacterium leprae* (*M. leprae*) or, more recently, *Mycobacterium lepromatosis*, an acid-fast bacillus with slow multiplication that cannot be cultured *in vitro* [1].

Leprosy has high prevalence in reported cases worldwide and is considered a neglected tropical and stigmatizing disease that has a significant negative impact on the lives of affected individuals. One of the goals of reducing disease burden and consequently improving the quality of life of affected individuals is to ensure adherence to multidrug therapy (MDT) [2].

The MDT has been the recommended treatment regimen for leprosy according to the World Health Organization’s (WHO). Since its adoption, significant progress has been made in reducing the prevalence of leprosy, decreasing it from over 5 million cases in the 1980s to 191,792 cases in 2024 in priority countries [2,3]. Ensuring timely treatment for all newly diagnosed cases based on the quality of systematic care for all patients with leprosy can help prevent relapse and consequently interrupt infection transmission [2].

MDT consists of a combination of antimicrobials (rifampicin, dapsone, and clofazimine) for all new cases and includes retreatment for disease relapse [4]. Since its introduction, the established treatment protocol for all individuals with leprosy has been divided into two distinct therapeutic regimens: one regimen with a combination of rifampicin, clofazimine, and dapsone for multibacillary (MB) cases and the other consisting only of rifampicin and dapsone for patients classified as paucibacillary (PB) [5,6].

To achieve the goal of eliminating leprosy as a public health problem, the World Health Organization (WHO) established changes in the treatment approach over the years of implementing the treatment strategy program. These changes include MDT until the bacilloscopy index (BI) becomes negative, particularly MDT with a fixed dose of 24 monthly doses and regular MDT with 12 monthly doses [5,6].

However, in 2018, the WHO implemented a new model for pharmacological leprosy treatment, known as Uniform Multidrug Therapy (MDT-U). This treatment protocol was adopted with a combination of the three previously mentioned drugs, which are now applied to all leprosy cases, regardless of the operational classification of the disease, maintaining a duration of six monthly doses for PB cases and 12 monthly doses for MB cases [4,7].

Although MDT is an important tool for controlling leprosy, failures in the therapeutic regimen commonly caused by diagnostic errors, inadequate treatment periods, irregular medication intake, and resistant strains tend to compromise the effectiveness of leprosy surveillance and trigger disease relapse [4,8–10].

Leprosy relapse is characterized by the reappearance of clinical activity of the disease (cutaneous and/or neurological) after treatment with standardized regimens; that is, when the patient is discharged as cured, they have completed the adopted therapeutic regimen. Relapse in leprosy cases is a significant issue, as it indicates a greater socioemotional and psychological burden for all individuals involved, primarily due to the increased likelihood of irreversible physical disability in these individuals [11–13]. Leprosy relapses can occur either early or late, typically between two to 15 years after treatment with MDT [14]. Early relapse may occur within a period of less than five years and is commonly associated with insufficient treatment, errors in the treatment’s operational classification, or irregular adherence to treatment. Late relapse can extend beyond five years and is possibly related to the persistence of bacilli or reinfection [10,15–17].

In 2024, it is estimated that 172,717 new cases of the disease were reported globally, corresponding to a detection rate of 21.1 per million population. The number of new cases detected worldwide was 5.5% lower than in 2023 (182,815). Brazil, India, and Indonesia each reported more than 10,000 new cases, together accounting for 79.8% of all new cases detected globally. While Indonesia registered a 2.2% increase in the number of new cases in 2024 compared to the previous year, decreases were reported in Brazil (2.8%) and India (6.4%) [3]. In 2024, 15,638 retreatment cases were reported, with Brazil, India, and Indonesia responsible for 83.5% of these cases. Relapses (4,488 cases) accounted for 28.7% of all retreatment cases. Of the 69 countries reporting relapses, Brazil, Ethiopia, India, and Indonesia accounted for 83.8% [3]. Relapse is an important indicator of leprosy service quality. The severity of leprosy relapse is not only related to bacillus infection, but also to the social, emotional, and psychological impacts involved in the context of the disease and the experiences of affected individuals (such as long treatment periods, leprosy reactions, pain, stigma, and disability) [13,15].

The present systematic review is relevant in this context. A preliminary search of PROSPERO, Medline, the Cochrane Database of Systematic Reviews, and JBI Evidence Synthesis did not identify any current or ongoing systematic review on the topic, highlighting a gap in knowledge regarding the synthesis of the estimated relapse prevalence.

This study aimed to synthesize the scientific evidence related to the estimation of the prevalence of leprosy relapse after MDT. Such knowledge could provide more robust information on the subject and contribute to planning strategies for clinical management and surveillance in the practice of care focused on this condition.

## Method

This was a systematic review of prevalence studies according to the JBI methodology [18–20]. The systematic review protocol was registered in the International Prospective Register of Systematic Reviews (PROSPERO: CRD42020177141. Access: https://www.crd.york.ac.uk/prospero/display_record.php?RecordID=177141).

### Inclusion criteria

The inclusion criteria were defined using the mnemonic PopCoCo (Population, Condition and Context). Pop: Patients of any age and sex diagnosed with leprosy relapsed after treatment with MDT. Co: Leprosy relapse was considered for patients diagnosed with relapse during a given period; that is, those who presented with clinical activity of the disease (cutaneous and/or neurological) after treatment with a standardized regimen (PB and MB), that has been discharged due to cure (treatment completed) [21–23]. The prevalence of relapse (proportion) was estimated (combined overall prevalence and its respective confidence interval (CI)), second MDT modality (24 doses until negative BI, 24 fixed doses, and 12 doses/regular), and country of study development [21–23]. The clinical characteristics of the participants were investigated in relation to the operational classification (PB/MB), time of relapse, bacilloscopy, histopathology, Physical Disability Score (DPD), and multidrug resistance to MDT components. The primary stage of interest was the overall estimate of relapse prevalence after MDT. Different periods of MDT implementation and study locations were also included. Co: Long-term studies at any geographic location and level of healthcare

### Exclusion criteria

Studies were excluded if they lacked data on relapse frequency in the study population, or if relapse occurred due to abandonment or irregular treatment, monotherapy, or alternative treatments.

### Types of studies

This review considered observational studies (cross-sectional, cohort, and case-control studies).

### Search strategy

An initial search limited to Medline was conducted using Medical Subject Headings (MeSH) terms and related keywords (multidrug therapy OR MDT OR leprosy OR “Mycobacterium leprae” OR “relapse” OR “risk factors” OR prevalence). This search was followed by an analysis of the words in the titles, abstracts, and indexing terms used to describe the studies.

A second search using all identified keywords and indexing terms was performed in the databases of the National Center for Biotechnology Information (NCBI) of the National Library of Medicine (NLM)/Medical Literature Analysis and Retrieval System Online (PubMed/Medline); Scopus; Web of Science (WoS); Embase; Latin American and Caribbean Health Sciences Literature (LILACS); Cumulative Index to Nursing and Allied Health Literature (CINAHL); and for unpublished studies (grey literature), The Caribbean Public Health Agency (CARPHA). Third, the reference lists of the studies selected during full-text reading were accessed.

The MeSH terms searched included multidrug therapy, relapse, leprosy, risk factors, and prevalence. Studies were identified from 1981 (following the recommendations for treatment from that period) to December 31, 2023. Language restrictions were not imposed.

A preliminary search was conducted in the PubMed/Medline database with the following query: ((((leprosy[MeSH Terms]) OR (mycobacterium leprae[MeSH Terms])) OR (multibacillary leprosy[MeSH Terms])) OR (paucibacillary leprosy[MeSH Terms])) (multidrug therapy [MeSH Terms])) OR (MDT [MeSH Terms])) AND (((((relapse[MeSH Terms]) OR (recurrence[MeSH Terms])) OR (risk factors[MeSH Terms])) OR (prevalence[MeSH Terms])). 1981, due to the treatment recommendation from this period, and up to 11 june, 2024. Using this strategy, we developed additional search strategies for each database Table 1.

**Table 1:** Descriptive summary of the search strategies used in the databases included in the systematic review.

Database	Strategy	Total
**Medline/PubMed**	((“multidrug therapy”[tw] OR MDT) AND leprosy[tw]) OR (((((leprosy[MeSH Terms]) OR (mycobacterium leprae[MeSH Terms])) OR (multibacillary leprosy[MeSH Terms])) OR (paucibacillary leprosy[MeSH Terms])) AND (((((relapse[MeSH Terms]) OR (recurrence[MeSH Terms])) OR (risk factors[MeSH Terms]))) OR (prevalence[MeSH Terms]))) Filters: from 1981/1/1 - 2024/06/11	2416
**Scopus**	(TITLE-ABS-KEY (mdt OR “multidrug therapy”) AND TITLE-ABS-KEY (leprosy OR “mycobacterium leprae” OR “multibacillary leprosy” OR “paucibacillary leprosy”) AND TITLE-ABS-KEY (relapse OR recurrence OR “risk factors” OR prevalence)) AND PUBYEAR > 1982 AND PUBYEAR < 2024	694
**Web Of Science**	“multidrug therapy” OR MDT (Topic) and (leprosy OR “mycobacterium leprae” OR “multibacillary leprosy” OR “paucibacillary leprosy”) (Topic) and (relapse OR recurrence OR “risk factors” OR prevalence) (Topic)	453
**Embase**	(‘polypharmacy’/exp OR ‘polypharmacy’ OR mdt OR ‘multidrug therapy’/exp OR ‘multidrug therapy’) AND (‘paucibacillary leprosy’/exp OR ‘paucibacillary leprosy’ OR ‘multibacillary leprosy’/exp OR ‘multibacillary leprosy’ OR ‘leprosy’/exp OR ‘leprosy’ OR ‘mycobacterium leprae’/exp OR ‘mycobacterium leprae’) AND (‘relapse’/exp OR relapse OR ‘recurrence’/exp OR recurrence OR ‘risk factors’/exp OR ‘risk factors’ OR ‘prevalence’/exp OR prevalence) AND [embase]/lim NOT ([embase]/lim AND [medline]	248
**Lilacs**	mdt OR MDT OR polychemotherapy AND (leprosy) OR (mycobacterium leprae) OR (paucibacillary leprosy) OR (multibacillary leprosy) AND (relapse) OR (recurrence) OR (“risk factor”) OR (prevalence) AND (db:(“ LILACS”))	58
**Cinhal**	TI (“multidrug therapy” OR MDT) OR AB (“multidrug therapy” OR MDT) OR SU (“multidrug therapy” OR MDT) AND OR AB (leprosy OR “mycobacterium leprae” OR “multibacillary leprosy” OR “paucibacillary leprosy”) OR SU (leprosy OR “mycobacterium leprae” OR “multibacillary leprosy” OR “paucibacillary leprosy”) OR (MH “Leprosy”) OR TI (leprosy OR “mycobacterium leprae” OR “multibacillary leprosy” OR “paucibacillary leprosy”) OR (MH “Recurrence”) OR TI (recurrence or relapse or reoccurrence) OR AB (recurrence or relapse or reoccurrence) OR SU (recurrence or relapse or reoccurrence) AND (MH “Risk Factors”) OR TI “Risk Factors” OR AB “Risk Factors” OR SU “Risk Factors” AND (MH “Prevalence”) OR TI Prevalence OR AB Prevalence OR SU Prevalence	39
**Carpha**	tw:(tw:(MDT OR MDT OR polychemotherapy AND leprosy OR mycobacterium leprae OR multibacillary leprosy OR paucibacillary leprosy AND relapse OR recurrence OR risk factor OR prevalence))	13

### Study selection

After implementing the search strategy in the selected databases, all identified citations were exported into the reference management software (Mendeley Reference Manager Online), and duplicates were removed. The titles and abstracts were screened for eligibility using the inclusion and exclusion criteria defined by two independent reviewers or a third reviewer to resolve discrepancies. Studies identified as potentially eligible, with or without abstracts, had their full texts retrieved and the details were imported into the JBI System for Unified Management, Assessment, and Information Review (JBI SUMARI) [19]. The Preferred Reporting Items for Systematic Reviews and Meta-Analyses (PRISMA) flow diagram was used [19,20].

### Methodological quality assessment

A methodological quality assessment of the included studies was performed using the JBI Critical Appraisal Checklist for studies reporting prevalence data. This checklist contains nine questions divided into three domains: participants (questions 1, 2, 4, and 9); outcome measurements (questions 6 and 7); and statistics (questions 3, 5, and 8). Studies were classified as high quality when the methods were appropriate across all 3 domains [24,25].

Two independent reviewers performed a critical assessment of the methodological quality. In case of disagreement, a third reviewer was consulted.

### Data extraction

The data were extracted by independent reviewers. A standardized data extraction table was used to collect and assess key information, including demographic characteristics, study location, sample size, number of leprosy and relapse cases, number of PB and MB cases at initial treatment and at relapse, according to the type of MDT regimen (24 doses until bacteriological index negativity, fixed 24-dose regimen, and standard 12-dose regimen). Additional variables included clinical characteristics and country in which the study was conducted. This process followed the essential elements outlined in the JBI SUMARI data-extraction tool for systematic reviews of prevalence studies.

### Data synthesis

Observational cohort studies were included in this meta-analysis. The inclusion of homogeneous studies made it possible to conduct a prevalence (proportion) relapse meta-analysis, estimating the global combined relapse and point prevalence according to the period of MDT institution and by country. A random-effects meta-analysis model was used to detail the overall combined relapse prevalence (global estimate) and subgroups. Heterogeneity among studies was assessed using the Higgins’ test (I²), which indicates the percentage of variation among studies by means of the confidence interval (CI95%) [19].

## Results

### Study selection

**Fig 1:**
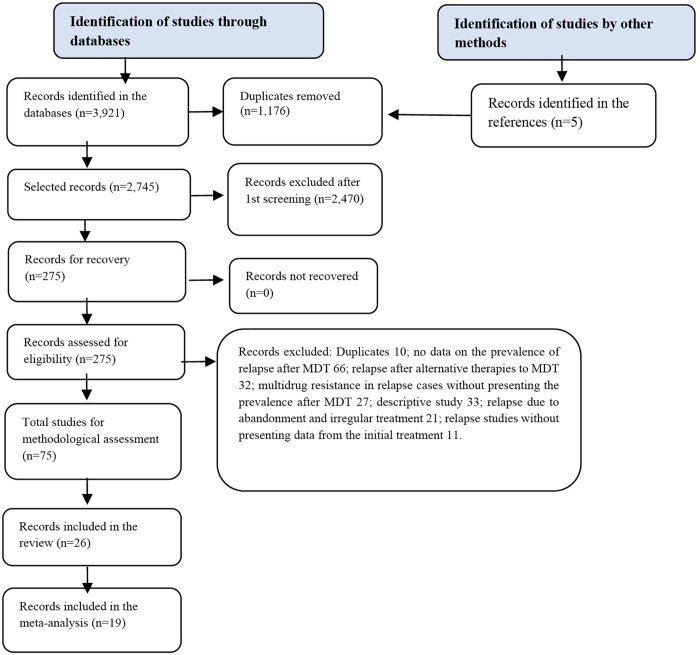
Flow diagram and summary of the systematic review stages according to the Preferred Reporting Items for Systematic Reviews and Meta-Analyses.

The search yielded a total of 3,921 studies identified from the databases and five records from other reference sources. Of these, 1,176 studies were excluded because they were duplicates, and 2,745 records were selected for title and abstract screening. Subsequently, 275 studies were selected for full-text review and methodological evaluation. A total of 26 studies were included in the review, and 19 were included in the meta-analysis, as presented in the PRISMA and JBI (Fig 1).

### Characteristics of the studies

Table 2 summarizes the main clinical findings of the studies included in the systematic review. Data were analyzed from 26 studies encompassing 71,385 patients previously treated with MDT, among whom 987 cases (1.38%) were documented as leprosy relapses. Of these, 428 (43.4%) were classified as MB, 367 (37.2%) as PB, and 192 (19.4%) were reported without specifying the operational classification at the time of relapse.

**Table 2:** Characteristics of the studies included in the systematic review, according to treatment and relapse in leprosy.

STUDY			INITIAL TREATMENT				RELAPSE		
Ref.	Author/Year	Country	Population	MB	PB	Treatment	Population	MB	PB
[26]	Grugni, et al. 1990	India	1509	0	1509	MDT UNTIL BI NEGATIVE	85	0	85
[27]	Boerrigter, et al. 1991	Malawi	484	0	484	MDT-R	12	0	12
[11]	Becx-Bleumink 1992	Ethiopia	5.444	2.379	3.065	MDT FD AND MDT UNTIL BI GOES NEGATIVE	58	24	34
[10]	Li, et al. 1997	China	235	235	0	MDT-FD	2	2	0
[28]	Chen, et al. 1999	China	47276	0	0	MDT FD AND MDT UNTIL BI GOES NEGATIVE	221	126	95
[29]	Girdhar, Girdhar, and Kumar 2000	India	561	561	0	MDT FD AND MDT UNTIL BI GOES NEGATIVE	32	0	0
[30]	Baohong 2001	Mali	35	18	17	MDT FD AND MDT UNTIL BI GOES NEGATIVE	7	0	0
[31]	Cellona, et al. 2003	India	500	500	0	MDT-FD	15	0	0
[32]	Ali, et al. 2005	India	3248	356	2892	MDT UNTIL BI NEGATIVE	58	3	55
[23]	Shen, et al. 2006	China	2282	2.282	0	MDT-FD	5	5	0
[24]	Poojabylaiah, et al. 2008	India	300	163	0	MDT-R, MDT FD AND MDT UNTIL BI GOES NEGATIVE	3	3	0
[33]	Balagon, et al. 2009	India	500	500	0	MDT-FD	23	0	0
[34]	Maghanoy, et al. 2011	Philippines	300	300	0	MDT-R	1	1	0
[35]	Guerrero 2012	Colombia	165	165	0	MDT FD AND MDT UNTIL BI GOES NEGATIVE	33	0	0
[36]	Kumar, Girdhar, and Girdhar 2012	India	599	0	599	MDT-R	35	0	0
[22]	Kumar, Girdhar, and Girdhar 2013	India	162	162	0	MDT-R	13	13	0
[37]	Prabu, et al. 2015	India	2177	902	1275	MDT-R	58	29	29
[38]	Nair, and Mathew 2017	India	389	285	104	MDT-R	39	31	8
[39]	Singhal, et al. 2018	India	78	78	0	MDT-R	7	7	0
[40]	Kaur 2020	India	144	132	12	MDT-R	10	7	3
[41]	Narang, et al. 2021	India	61	44	17	MDT-R	37	0	0
[42]	Nery, et al. 2021	Brazil	713	713	0	MDT-R	10	0	0
[43]	Rajkumar, et al. 2021	India	1948	0	0	MDT-R	69	31	38
[44]	Rosa 2021	Brazil	835	0	0	MDT-R	23	21	2
[45]	Mahajan, et al. 2021	India	381	308	73	MDT-R	5	4	1
[46]	Nascimento, et al. 2022	Brazil	1059	0	0	MDT-R AND MDT FD	126	121	5
			**71385**	**10083**	**10047**		**987**	**428**	**367**

MB: Multibacillary; PB: Paucibacillary; BI: Bacillary Index; MDT-R: Regular Multidrug Therapy; MDT FD: Fixed-Dose Multidrug Therapy; MDT until Negative: Multidrug Therapy until Bacilloscopy Negativity.

The findings of this systematic review revealed considerable variability in the prevalence of leprosy relapse among patients treated with different MDT regimens, particularly among those classified as MB.

Of the studies analyzed, thirteen studies (50%) investigated relapse in patients treated with the standard MDT regimen (MDT-R), which consisted of 12 doses for MB cases and 6 doses for PB cases. Four studies (15%) assessed the occurrence of relapse in individuals treated with a fixed-duration 24-month MDT regimen (MDT-FD). Additionally, five studies (19%) examined an extended MDT approach, which began with a fixed 24-month duration and continued until bacillary index (BI) negativation (MDT-FD until negativation). Two studies (8%) exclusively evaluated MDT administered until BI negativation (MDT-until negativation). One study (4%) investigated relapse in patients treated with both MDT-R and MDT-FD regimens, and another (4%) included patients treated with three regimens: MDT-FD for 24 doses until BI negativation, MDT-FD fixed for 24 doses, and MDT-R for 12 doses. Most of the studies were conducted in India (n = 15; 58%), followed by Brazil (n = 3; 11%), China (n = 3; 11%), and other countries, including the Philippines, Colombia, Mali, Ethiopia, and Malawi, which together accounted for five studies (20%).

The synthesis of the main clinical findings included in the review (Table 3) identified a higher prevalence of leprosy relapse among male individuals and those with a mean age of more than 30 years. The studies demonstrated that the time to relapse following initial treatment with MDT was five years or more after clinical cure, with higher prevalence observed among patients treated for MB forms. A higher frequency of relapse cases with positive bacilloscopy results was noted, with the average BI ranging from 1+ to 4 + . Histopathological examinations were conducted in 14 studies, with borderline lepromatous and lepromatous-lepromatous clinical forms being the most prevalent.

**Table 3:** Descriptive summary of the main clinical findings from the studies included in the systematic review.

Nº	Author/Year	Country	Initial Population	Population at Relapse	Sex	Mean Age	BI	Histopathology	DPD	MDT resistance	Main considerations
[26]	Grugni, et al. 1990	India	1509	85	Na	NA	NI	Indeterminate: 3% TT: 25% BB: 54% Pure neurite: 3%	NA	NA	A relapse rate of 5.63% was estimated; 79% of patients recurred with skin lesions, 15% with neuritis and 6% with a mixed pattern.
[27]	Boerrigter, et al. 1991	Malawi	484	12	Female- 33% Male- 67%	Mean age: (0–14): 15.0% (15-29): 4.2% (30-44): 7.3% (>45): 3.6%	NI	TT: 8.33% TT/BT: 8.33% BT: 58.33% BB: 25%	NI	NA	A relative lack of cell-mediated immunity, by number of lesions, clinical classification and lepro min test results, and poor compliance with the dapsone component of MDT, was associated with an increased risk of relapse.
[11]	Becx-Bleumink 1992	Ethiopia	5.444	58	Na	NA	NC	NC	NA	NA	Relapse did not vary significantly from year to year (p = 0.25).
[10]	Li, et al. 1997	China	235	2	Female- 23% Male- 77%	Mean age: 39.5	BI 1–4 + : 81,25% BI -: 18,75%	LL: 25% BL: 37.5% BT: 18.75% BB: 18.75%	NA	NA	The mean relapse rate of patients treated for MB: 5/ 26,804 = 0.19/ 1000 people/year.
[28]	Chen, et al. 1999	China	47276	221	Na	NA	NI	NA	NA	NA	The relapse rate in patients treated with MDT-PB was significantly higher than in patients treated with MDT-MB. The risk of relapse is higher in patients with MDT-PB in the first 8 years (p < 0.01). With PB leprosy, more than half (55.8%) relapsed with MB leprosy (p < 0.01).
[29]	Girdhar, Girdhar e Kumar 2000	India	561	32	Na	NA	NA	NA	NA	NA	The relapse rate was therefore significantly higher in patients with BI 2:4 (P = 0.0002). Of the total number of relapses, 17 occurred in the first 3 years of follow-up and the remaining three more than 5 years after stopping treatment.
[30]	Baohong 2001	Mali	35	7	Na	NA	NA	NA	NA	NA	All seven relapses were observed among the 18 patients who had an initial bacterial index (BI), i.e., mean BI before MDT, of 2:4.0, whereas no relapse was detected among the 17 patients whose initial BI was < 4.0.6. This indicates a correlation between relapse and BI before MDT, and a high initial BI of 2:4.0 among patients with MB leprosy.
[31]	Cellona, et al. 2003	India	500	15	Female- 25% Male- 75%	Mean age: 32.5	BI > 4 + : 36% BI < 4 + : 64%	BL: 60% LL: 40%	NA	NA	The study showed that in patients followed up for ≥12 years and who had an initial BI ≥ 2.7 + , the relapse rate was 13% (13/98) vs. 0% (0/44) in those with an initial BI < 2.7.
[32]	Ali, et al. 2005	India	3248	58	PB: Female- 43% Male- 57% MB: Not Evaluated.	NC	NI	NI	PB: D1-2: 56.36% MB: Not evaluated	^NA^	Relapse was recorded up to a maximum of 16 years after fixed-duration treatment.
[23]	Shen, et al. 2006	China	2282	5	Male-90% Female- 10%	NA	BI 1.80–5,33 + : 100%	NA	NA	NA	Patients with relapse occurred 48–158 months after the end of MDT. The relapse rate of MB patients treated at 24 months was observed to be very low after long-term follow-up.
[24]	Poojabylaiah, et al. 2008	India	300	3	Male 100%	Mean age: 45.3	BI 2 + : 33.33% BI3 + : 33.33% BI4 + : 33.33%	LL100%	NA	NA	All relapse cases were males with LL disease. Cases of relapse occurred in patients who received MDT until smear negativity, when compared to patients who received fixed-duration therapy.
[33]	Balagon, et al. 2009	India	500	23	Female- 25% Male- 75%	Mean age: 32.5	BI 4.0 + : 36%	BB 9% BL 26% LL 47.80% Histoid 17.40%	NA	Sensitivity to rifampicin and clofazimine	Relapses had an approximate ratio of 6–1 between men and women. The relapse rate peaked at ages 11 and 12.
[34]	Maghanoy, et al. 2011	Philippines	300	1	Male-100%	Mean age: 30	BI2 + : 0.6%	BL: 100%	NA	NI	The study suggests that medication resistance is less likely due to the negativity of the patient’s smear before relapse.
[35]	Guerrero 2012	Colombia	165	33	Male 19 Female 14	Mean age: 39	14 BI + cases	NA	D1 ≥ : 36.40%	NA	Relapses were diagnosed after receiving between 25 and 48 doses of regular treatment, which may suggest the need to evaluate other regimens for those who persist with positive smears after 24 doses.
[36]	Kumar, Girdhar e Girdhar 2012	India	599	35	Female- 54.7% Male- 45.3%	Mean age: 34.2	BI 1 + : 0.3%	BT: 84.3%	D0- 79% D1: 40%	NA	Physical disability was 2.2% and varied significantly according to age and nerve thickening.
[22]	Kumar, Girdhar e Girdhar 2013	India	162	13	> Male	Mean age: 46.1	BI + : 15.4% BI-: 53.84% Not evaluated: 30.80%	BT/BTR: 38.5% BB/BBR: 46.20% BL/LL/Neuritic: 15.4%	D0: 15.4% D1-2: 38.5% D > 3:46.20%	NA	Predominance in 34 years olds, males and among BB patients and BI positive.
[37]	Prabu, et al. 2015	India	2177	58	Female- 31% Male- 69%	Mean age: 34	NI	NA	D0- 36.40% D1- 27.30% D2- 36.4%	NI	Predominance of cases in MB. Men had significantly higher relapses than women.
[38]	Nair e Mathew 2017	India	389	39	Female- 15.39% Male- 84.61%	Mean age: 46.82	BI 0–4 + :35.90%	Indeterminate 7.70% Pure neuritic 7.70% BT 46.15% BB 2.56% BL 10.26% LL 20.52% Histoid 5.13%	D0 ≥ : 35.89%	NI	Relapse was more common in leprosy cases with an initially positive bacilloscopy. At relapse, 35.90% had BI+ again. 57.14% of relapse cases were treated with fixed-duration treatment for one year.
[39]	Singhal, et al. 2018	India	78	7	Na	Mean age: 37.4	NA	NA	D1: 8% D2: 20% D1-2: 4%	NA	Of the 25 cases, 5 showed signs of activity and relapse in the form of the appearance of new nerve involvement appearance (2; 8%), of a new lesion (5; 20%) and of a new deformity (1; 4%).
[40]	Kaur 2020	India	144	10	Na	Mean age: Female: 36.78 to 15.79/ Male: 36.59 to 16.58	NA	MB: LL:5/B L:1/BB1	NA	NA	Eighty percent of relapses were observed within 5 years of RFT showing early occurrence of relapse. In contrast, our study showed a higher relapse rate of 6.94%.
[41]	Narang, et al. 2021	India	61	37	Male 44% Female 17%	Mean age: 35.3	BI medium 2.42+	NA	NA	dapsone; rifampicin; ofloxacin; multi drug resistance	Rates of drug resistance was found to be 5.4% (2/37) for dapsone, 10.8% (4/37) for rifampicin and 2.7% (1/37) for ofloxacin amongst cases of relapse. Amongst those with c/rENL, rates of resistance were 12.5% (3/24) and 8.3% (2/24) for dapsone and rifampicin respectively.
[42]	Nery, et al. 2021	Brazil	713	10	Female- 20% Male- 80%	Mean age: 40.2	BI 00–01 + :5 BI 02–04:4 BI > 5:1	ND:2 BB: 1 BL:3 LL:4	NI	NI	The study showed that although high bacillary load was considered a risk factor for relapse, no significant data was found to reinforce the hypothesis.
[43]	Rajkumar, et al. 2021	India	1948	69	Female- 31% Male- 68%	Mean age: 34.5	BI = 0,25 a 4,5 (9,4%)	NA	D 0 ≥ : 30.9%	NA	The findings show a mean relapse occurrence time of 5 years, with the risk of relapse decreasing over the years.
[44]	Rosa, 2021	Brazil	835	23	Male 15% Female 8%	Mean age 24.5 e 26.3	NA	NA	NA	dapsone; rifampicin;	16 (43.24%) presented medication resistance variants. Multidrug resistance to rifampicin and dapsone was observed in 8 relapses and 4 new cases.
[45]	Mahajan, et al. 2021	India	381	5	Na	Mean age: 21 and 60	NA	BL:8 LL:3 e TT:2	NA	NA	Seven (1.8%) MB leprosy patients required prolonged treatment for 18–24 months due to high BI. Thirteen (3.25%) of the patients were HD relapsers, of whom a maximum of eight were BL HD followed by LL HD three (0.75%) and TT HD two (0.5%).
[46]	Nascimento, et al. 2022	Brazil	1059	126	Female- 46% Male- 54%	Mean age: 49.5	BI 0–6 + : LL and BL and MB negative bacillary	Neural 8 6.3 BT 51 40.5 BB 9 7.1 BL 11 8.7 LL 47 37.3	D0 43.7% D1 26.2% D2 25.4%	Rifampicin and dapsone	A study identified a higher prevalence of relapse among MB cases, and the presence of resistance to the medications that make up MDT, showing cases of resistance to all three medications. Taking 24 doses of MDT was associated with a better prognosis in relation to relapse over time, compared to 6 or 12 doses of MDT therapy. The majority of relapse cases were classified as multbacillary (96.03%; 121/126).

MB: Multibacillary; PB: Paucibacillary; TT: Tuberculoid Tuberculoid; BT: Borderline Tuberculoid; BB: Borderline Borderline; BL: Borderline Lepromatous; LL:Lepromatous Lepromatous; MDT-R: Regular Multidrug Therapy; MDT FD: Fixed-Dose Multidrug Therapy; MDT until Negative: Multidrug Therapy until Bacilloscopy Negativity; NA - Not Assessed; NC - Not Clear; NI - Not Identified; Female - Female; Male - Male; BI - Bacillary Index; Histopathology; DPD - Degree of Physical Disability.

### Summary of the main results of the meta-analysis

The random-effects meta-analysis included 19 studies (combined sample of 68,369 participants) and was stratified by subgroups according to the country in which the study was conducted and the type of treatment administered. The estimated prevalence of relapse ranges from 0 to 10% depending on the geographic location and treatment modality.

**Fig 2:**
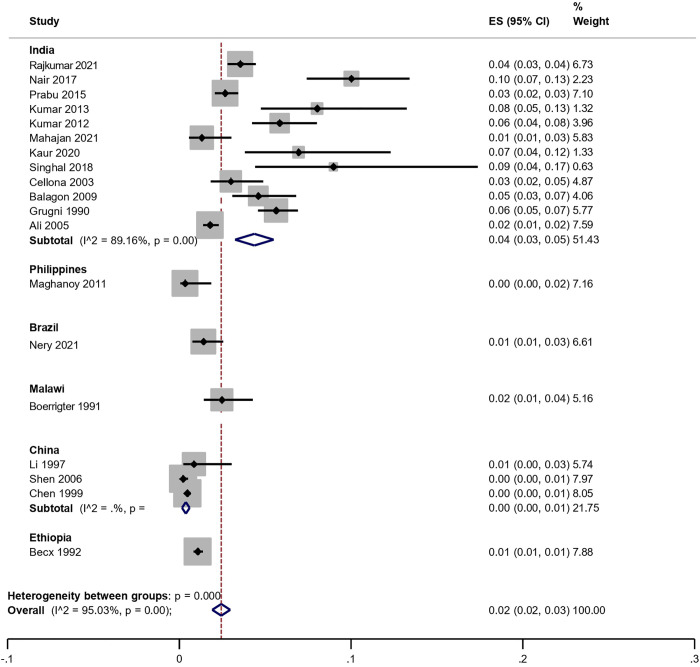
Forest plot for a random-effect meta-analysis of global prevalence of leprosy relapse, according to the country of study.

The meta-analysis stratified by country revealed an overall pooled proportion of leprosy relapse of 0.02 (95% CI: 0.02–0.03), indicating substantial heterogeneity across studies (I² = 95.03%; p < 0.001). The relapse prevalence based on geographic region showed the highest pooled point estimate in India (12 studies; n = 11,635 participants), with a relapse proportion of 4% (95% CI: 0.03–0.05), contributing a weight of 51.43% to the meta-analysis. (Table 4 and Fig 2).

**Table 4:** Synthesis of meta-analysis study data according to prevalence estimated by country.

Country	Relapse Rate (%)	Confidence Interval (IC95%)	Weight (%)
**India**	0,04	0,03-0,05	51,43%
**Philippines**	0	0,00-0,02	7,16%
**Brazil**	0,01	0,01-0,03	6,61%
**Malawi**	0,02	0,01-0,04	5,16%
**China**	0	0,00-0,01	21,75%
**Ethiopia**	0,01	0,01-0,01	7,88%
**Overall**	0,02	0,02-0,03	100%

China presented the lowest relapse proportion, with a point estimate of 0.00 (95% CI: 0.00–0.01; three studies, n = 2,517 participants), contributing 21.75% of the overall analysis weight. This was followed by the Philippines, with a prevalence estimate of 0.00 (95% CI: 0.00–0.02; one study, n = 300 participants), contributing 7.16% of the total weight. Brazil, Ethiopia, and Malawi showed relapse estimates ranging from 1% to 2%, each represented by a single study: Brazil (95% CI: 0.01–0.03; weight: 6.61%), Ethiopia (95% CI: 0.01–0.01; weight: 7.88%), and Malawi (95% CI: 0.01–0.04; weight: 5.16%) (Table 4 and Fig 2).

**Fig 3:**
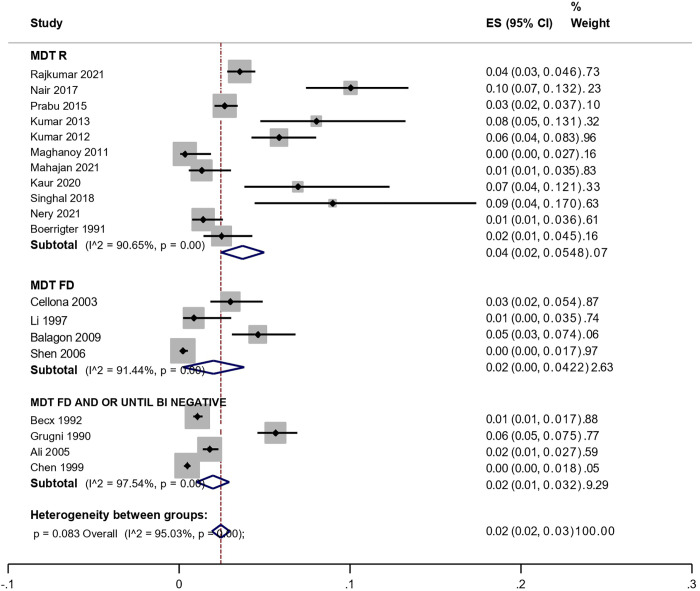
Forest plot for a random-effect meta-analysis of global prevalence of leprosy relapse, according to multidrug therapy strategies.

The analysis stratified by treatment type revealed a higher pooled point estimate of leprosy relapse among individuals treated with MDT-R (0.04; 95% CI: 0.02–0.05; weight: 48.07%) when compared to those treated with MDT-FD or MDT-FD until BI negativation (Table 5 and Fig 3). This analysis also demonstrated significant heterogeneity across studies (I² = 95.03%; p < 0.001). Across the full set of studies, no statistically significant differences were observed in relapse prevalence estimates (p = 0.083) (Table 4 and Fig 2).

**Table 5:** Synthesis of meta-analysis study data according to prevalence estimated by type of treatment.

Treatment	Relapse Proportion (%)	Confidence Interval (CI 95%)	Weight (%)
**MDT-R**	0,04	0,02–0,05	48,07%
**MDT-FD**	0,02	0,00–0,04	22,63%
**MDT-FD until BI negativation**	0,02	0,01–0,03	29,29%
**Overall**	0,02	0,02–0,03	100%

## Discussion

This systematic review assessed the prevalence of leprosy relapse after MDT and included 26 studies with 71,385 participants. Among those who experienced relapse, the majority were male, economically active, and classified as having MB with a high bacillary load and a higher grade of physical disability.

Of the 19 studies included in the meta-analysis involving 68,369 participants, the majority were conducted in India and collectively indicated a pooled relapse prevalence of 4%, in contrast to the lower rates observed in studies from China. Subgroup analysis by treatment regimen also revealed a higher pooled prevalence of relapse among participants treated with the MDT-R regimen than among those treated with MDT-FD and MDT-FD until BI negativation. However, no statistically significant differences were found among the pooled relapse estimates across treatment regimens, and substantial heterogeneity was observed across studies. Overall, the relapse prevalence varied depending on the study design, follow-up duration, and diagnostic criteria.

Despite the positive effects of MDT, its effectiveness in preventing relapses remains limited. The persistence of viable bacilli following treatment, leprosy reactions, and silent neuritis represent an ongoing challenge in the clinical management of these patients. Such complexities, particularly in MB cases, underscore the need to strengthen surveillance and ensure therapeutic adherence [7,17,47,48].

In this context, the use of biomarkers to monitor therapeutic responses is promising. However, large-scale validation is still lacking [49]. Furthermore, discharge criteria based solely on treatment duration should be considered. Therefore, leprosy management approaches should extend beyond the administration of MDT by incorporating health education, strengthening epidemiological surveillance, and physical rehabilitation [50,51].

The evidence of drug resistance to current MDT regimens, as reported in some of the studies included in this review, highlights the concern about an increased risk of relapse, particularly in settings with low treatment adherence or inadequate follow-up of all diagnosed patients [52,53].

From a pathophysiological perspective, *M. leprae* exhibits an extremely slow metabolism, allowing the persistence of viable forms even after completion of MDT [1]. This phenomenon, known as “subclinical persistence,” is considered one of the main biological causes of late relapse [1,5,6]. The risk of relapse depends not only on the therapeutic regimen but also on the host’s immune response, initial bacillary load, and the quality of treatment adherence [8,10,13]. The significance of these factors is supported by the predominant profile of relapse cases identified in this review primarily male patients diagnosed with MB forms [17].

The results of this meta-analysis revealed differences in relapse estimates when subgroups were analyzed according to geographic location and according to implementation. This synthesis indicated high heterogeneity across the studies and no significant association with relapse occurrence when evaluating the overall pooled estimates. However, upon interpreting the results, a higher relapse estimate was identified in India, the global epicenter of leprosy, accounting for approximately 60% of the global disease burden [3,21,43]. These findings may play a decisive role in advancing our understanding of relapse after MDT.

The predominance of studies conducted in India, as reported in this review, may be an influencing factor in the results yet also plays a decisive role in contributing to the occurrence of leprosy relapse. Factors such as passive surveillance, limited access to healthcare in rural areas, and low rate of contact tracing may be associated with this outcome [11,26].

On the other hand, the geographic locations represented by the Philippines, Brazil, Malawi, and Ethiopia showed relapse estimates ranging from zero to 2%, although the weight of each study in the meta-analysis was negligible. This highlights the need for greater investments in research on leprosy relapse to better assess the effectiveness of control programs in these countries. Reports from these regions have described operational challenges in case follow-up, irregular treatment, and delayed diagnosis, all of which contribute to relapse. These conditions justify increased research funding to support more effective strategies for the clinical management of affected patients [3,11,54].

The results also indicated lower relapse estimates in China, as observed in three studies. In China, active case detection, strengthening of the primary healthcare system, and use of risk-based surveillance strategies have been identified as key measures for reducing the incidence and preventing relapse, which may explain the low relapse rates reported in this review [53,55]. Despite effective national-level control, leprosy persists in specific areas. Higher spatiotemporal disease patterns, historical disease clustering, and rural-to-urban migration contributed to elevated relapse estimates in these regions [56].

Analysis of the studies included in this review highlights the heterogeneity of the therapeutic regimens adopted for leprosy treatment, reflecting both the evolution of clinical guidelines over time and variations in clinical practice across different geographic and institutional settings. A greater number of studies have focused on evaluating relapse among patients treated with the MDT-R regimen, with 13 studies (50%) exclusively addressing this regimen, consisting of 12 monthly doses for MB cases and six doses for PB cases, as recommended by the WHO [57].

These findings may be explained by the widespread adoption of the MDT-R regimen as a standard treatment since the 1980s, which has made it a reference for clinical and epidemiological studies assessing the long-term effectiveness and outcomes of leprosy treatment. Furthermore, the consolidation of this regimen within national leprosy control programs has contributed to its predominance in literature.

Although the MDT-R regimen showed a higher estimated relapse rate than the MDT-FD and MDT-FD regimens until the negativation regimens, the differences between subgroups did not support a definitive association. These findings suggest that factors such as individual patient characteristics, the quality of treatment adherence, and the capacity of healthcare service delivery may have influenced the outcomes observed in this review [47,58,59].

Four studies (15%) investigated relapse in patients treated with the MDT-FD regimen, an approach primarily adopted in contexts with high bacillary burden, especially decades before the widespread implementation of MDT-R. This regimen was, in certain situations, considered more effective particularly in reducing bacillary load and preventing relapse, especially among MB patients with high initial bacilloscopic indices [15,17,60].

Additionally, five studies (19%) evaluated extended MDT regimens consisting of a fixed 24-month duration combined with continued treatment until BI negativation. This strategy is commonly adopted in referral centers and specialized services where ongoing bacteriological monitoring is feasible. The technical rationale for this approach is based on the premise that the persistence of viable *M. leprae* after completion of treatment may be a key determinant of relapse, particularly in patients with high initial bacillary indices [61,62].

Two studies (8%) exclusively investigated the effectiveness of MDT administered until BI negativation without a predetermined treatment duration. Although this approach is supported by laboratory evidence and long-term follow-up studies, its large-scale implementation is limited by laboratory requirements, cost, and the need for infrastructure to support regular monitoring of BI, which may explain the limited number of publications on this regimen [63,64].

Finally, one study (4%) compared the MDT-R and MDT-FD regimens, whereas another (4%) evaluated multiple therapeutic protocols, including MDT-FD with 24 doses until BI negativation, fixed 24-dose MDT-FD, and the standard 12-dose MDT-R regimen. These studies provide valuable comparative evidence regarding the relative effectiveness of different treatment strategies, although they remain scarce in literature. The limited number of such studies may be attributed to methodological complexity, the extended follow-up period required to assess relapse, and variability in relapse definition and detection across study centers.

Therefore, the findings of this review predominantly focus on the MDT-R regimen owing to its widespread implementation and global standardization. The low frequency of studies investigating alternative or individualized regimens underscores the need for further research that systematically explores relapse-related outcomes considering the diversity of clinical, epidemiological, and operational contexts.

Although MDT has revolutionized leprosy treatment, relapse may still occur due to reactivation of latent infection, reinfection, or drug resistance [9,12,13,15]. The attempts to modify therapeutic regimens since the introduction of MDT, as highlighted in this review, reflect the need for a more comprehensive care model that emphasizes early detection, management of leprosy reactions, post-treatment monitoring, and diagnostic innovation. Current discharge criteria, based solely on treatment duration without bacteriological confirmation—particularly in complex cases with high bacillary loads—should be reconsidered in different geographic contexts [22,23,46,65,66]. However, advances such as the decentralization of treatment and the implementation of strategies such as contact monitoring have proven to be key determinants of improved leprosy control, helping minimize the potential for relapse.

## Limitations

Our study has several limitations. Factors such as high heterogeneity among the included studies, lack of standardization in the diagnosis of relapse and cure, and incomplete data on the clinical characteristics of the participants may have affected the precise estimation of prevalence. Nevertheless, subgroup analyses by country and modality of MDT implementation strategies enhanced the interpretability and robustness of the findings.

## Conclusion

A higher prevalence of relapse was observed among MB patients with high bacillary loads and a greater frequency of established physical disability, predominantly affecting male patients of an economically productive age. The pooled relapse prevalence estimate was highest in India, recognized as the global epicenter of reported leprosy cases, and contributed to the largest number of studies included in this review. Among the therapeutic regimens analyzed, a higher prevalence of relapse was associated with the 12-dose MDT strategy than with the 24-dose fixed-duration regimen, which continued until bacillary negativation. These findings suggest that individual patient characteristics, quality of treatment adherence, and the capacity for healthcare services may have influenced the outcomes observed in this review.

Monitoring patients during treatment, along with clinical, histological, serological, and molecular assessments of their contacts, may help reduce the risk of relapse. Future studies should be promoted by policymakers to evaluate the implementation of uniform therapeutic regimens, particularly for complex cases, such as MB patients with a high disease burden, in varied geographic settings.

## Supporting information

S1 PRISMA Checklist PRISMA Checklist. (DOCX)
